# A Population-Based Study of the Behavioral and Emotional Adjustment of Older Siblings of Children with and without Intellectual Disability

**DOI:** 10.1007/s10802-018-00510-5

**Published:** 2019-02-04

**Authors:** Nikita K. Hayden, Richard P. Hastings, Vasiliki Totsika, Emma Langley

**Affiliations:** 1 Centre for Educational Development, Appraisal and Research, University of Warwick, Coventry, CV4 7AL UK; 2 Centre for Developmental Psychiatry and Psychology, Department of Psychiatry, Monash University, Melbourne, Australia; 3 Division of Psychiatry, University College London, London, UK

**Keywords:** Siblings, Behavioral and emotional adjustment, Intellectual disability, Population sample, Families

## Abstract

This is the first study on the behavioral and emotional adjustment of siblings of children with intellectual disabilities (ID) to use a population-based sample, from the third wave of the Millennium Cohort Study (MCS); a UK longitudinal birth cohort study. We examined differences between nearest-in-age older siblings (age 5–15) of MCS children (likely mainly with mild to moderate ID) identified with ID (*n* = 257 siblings) or not (*n* = 7246 siblings). The Strengths and Difficulties Questionnaire (SDQ) measured all children’s adjustment. For SDQ total problems, 13.9% of siblings of children with ID and 8.9% of siblings of children without had elevated scores (*OR* 1.65; 95% CI 1.04, 2.62; *p* = 0.031). Similar group differences were found for SDQ peer and conduct problems. In logistic regression models, variables consistently associated with older sibling adjustment were: adjustment of the MCS cohort child, older sibling being male, family socio-economic position, primary carer psychological distress, and being from a single parent household. The ID grouping variable was no longer associated with adjustment for all SDQ domains, except siblings of children with ID were less likely to be identified as hyperactive (*OR* 0.30; 95% CI 0.10, 0.87; *p* = 0.027). Some older siblings of children with ID may be at additional risk for behavioral and emotional problems. Group differences were related mainly to social and family contextual factors. Future longitudinal research should address developmental pathways by which children with ID may affect sibling adjustment.

## Introduction

Although there is a range of existing research exploring developmental outcomes for children who have a brother or sister with a disability, including intellectual disability (ID), current research is both inconsistent and contradictory in answering whether these siblings of children with ID are at an increased risk of poorer outcomes – as might be predicted from family systems theory (Kovshoff et al. 2017). For example, an early meta-analysis found that siblings of children with ID had more psychological and social problems than comparison groups, although these group differences were small (Rossiter and Sharpe 2001). In more recent studies, researchers have reported similar relatively small group differences but also considerable variability in outcomes. A number of studies have identified more behavioral and emotional problems in siblings of children with disability compared to other children or to normative samples (Hastings 2003; Verté et al. 2003; Ross and Cuskelly 2006; Orsmond and Seltzer 2007; Goudie et al. 2013). Other researchers found little or no group difference (Cuskelly and Gunn 2006; Hastings 2007; Howlin et al. 2015).

There is a distinct lack of representative population-based studies in this area. The strength of such studies is they are less affected by referral or self-referral biases and may allow conclusions about the whole population of siblings, or the whole population of siblings of children with ID. We found three population based studies of siblings of children with disability, but no population-based studies focusing on siblings of children with ID specifically. Focusing on ID specifically is important since different disability profiles or diagnoses have been shown to be associated with varying impact on the family system including siblings and parents (Hastings 2016). In addition, key putative risk factors for sibling outcomes such as the behavioral and emotional adjustment of the disabled child and parental psychological distress are more prevalent in families of children with ID compared to other families of children with other disabilities (Hastings 2016).

Goudie et al. (2013), analyzing data from the USA Medical Expenditure Panel Survey, identified 245 siblings of children with disability and 6564 siblings of children without disability. Disability in this study included those with cognitive disabilities, physical disabilities and chronic conditions. Goudie et al. (2013) found siblings of children with disability had more social problems, problems with behavior, and problems in school. For example, siblings of children with disability were 2.77 times more likely to have significant levels of problem behavior.

Emerson and Giallo (2014), using a nationally representative group of children in Australia, explored the psychological well-being and adjustment of siblings of children also with a broad range of disabilities. There were 7636 children included in the analysis who were living with at least one sibling for both study waves included in the research that did not have a brother or sister with a known disability, and 286 children that had a brother or sister with disability. Emerson and Giallo (2014) initially found that siblings of children with disability had lower well-being than the group of siblings without a disabled brother or sister in some, but not all adjustment areas. However, once analyses controlled for the effects of socio-economic deprivation and other associated hardships, the small group differences in well-being were no longer statistically significant. Thus, the putative impact of child disability on sibling outcomes may have been mediated via socio-economic deprivation or explained directly by exposure to poverty. Such competing hypotheses could not be tested in the context of the cross-sectional methods used.

Neely-Barnes and Graff’s (2011) study in the USA using national health data showed similar results when measuring psychological outcomes of siblings of children with disabilities. Neely-Barnes and Graff (2011) identified 373 siblings of a child with a disability and 3790 eligible siblings of children without a disability. Between-group differences were non-significant once 12 additional factors were controlled. Again, co-occurring risk factors such as low income appeared to explain the association between child disability and sibling outcomes. However, causal effects could not be established within this cross-sectional design.

The Goudie et al. (2013), Emerson and Giallo (2014) and Neely-Barnes and Graff (2011) studies explored disabilities defined broadly and did not focus specifically on ID. The latter two studies do indicate the importance of exploring alternative factors that may affect sibling outcomes either directly or that may be associated with having a disabled brother or sister. In particular, socio-economic deprivation and economic factors affecting families are key variables. Emerson and Giallo (2014) also included maternal mental health in the variables they examined (as would likely be important from a family systems perspective), but the other population-based studies did not. Thus, there is still no population-based analysis of siblings of children with ID from a dataset that also allows the exploration of the impact of a number of correlates of sibling outcomes and examined for their independent effects. Furthermore, the three existing population based studies differ in their findings. Therefore, further work is needed not only with an ID focus, but to understand more about correlates of sibling behavioral and emotional adjustment.

Factors other than socio-economic variables might also affect siblings’ behavioral and emotional adjustment. These include: the age and sex of the sibling (Hastings 2003; Verté et al. 2003; Cuskelly and Gunn 2006; Orsmond et al. 2009; Petalas et al. 2009; Walton 2016); the sex composition of sibling dyads (Cuskelly and Gunn 2006; Ivey and Barnard-Brak 2009); birth interval (Martin and Horriat 2012); the number of brothers and sisters in the household (Burke et al. 2012; Goudie et al. 2013; Walton 2016), and whether the family is a single parent/carer household (Deater-Deckard and Dunn 2002; Kelly et al. 2009; McHale et al. 2012).

More importantly, in addition to socio-demographic factors, a family systems perspective on outcomes for siblings of children with disability (Kovshoff et al. 2017) suggests that the well-being of other family members is likely to affect sibling psychological adjustment. Consistent with this systems perspective, a number of studies have shown associations between maternal psychological distress and sibling adjustment (Quintero and McIntyre 2010; Petalas et al. 2012), and between the behavioral and emotional problems of children with ID and their siblings’ psychological adjustment (Hastings 2007). Existing population-based studies of siblings of disabled children have not examined the independent associations of additional putative risk factors such as parental psychological distress and the siblings’ brother or sister’s behavioral and emotional adjustment in addition to a range of socio-economic and demographic factors.

The aims of the present study were, therefore, to: (i) explore if there were group differences in behavioral and emotional adjustment for siblings of children with and without ID in a nationally representative sample, and (ii) to explore, if there were differences, which correlates identified from the existing literature were associated with sibling adjustment. These two research questions extend existing literature through the focus specifically on ID, in a population-representative sample, and incorporating a wider range of known correlates of sibling adjustment.

## Method

This study is a secondary analysis of data from the third wave of the UK’s ongoing Millennium Cohort Study (MCS3; “Millennium Cohort Study: Third Survey,” 2017). MCS is a longitudinal birth cohort study following the lives of 19,000 children born in 2000–2001 (MCS 2017), with MCS3 following up with cohort member children at age five years. Cohort children are identified through Child Benefit Records – a non-means tested state universal benefit with a very high uptake among UK families with children at the time of the MCS inception. Cohort member children were randomly selected from those children eligible to receive Child Benefit, living across the UK, at age nine months, and born between September 2000 and August 2001 (Plewis 2007). A two-stage stratification was followed to ensure that a nationally representative sample with adequate representation from ethnic minority and disadvantaged children was achieved. Weights were subsequently developed to account for the MCS sample design (Hansen 2012). In the present study, to ensure the sample is a nationally representative sample, data were analyzed with weightings through complex samples procedures (Jones and Ketende 2010).

In the present study, the data analyzed were collected from the primary respondent. This was the mother (biological or adoptive) in 99.8% (weighted) of families. The remaining primary respondents were also included in the analysis and included fathers (natural, adoptive or step) as well as other extended family members. Primary respondents will be referred to as primary carers throughout this paper. Data about the MCS cohort members’ older siblings (but not younger siblings, given the young age of the cohort children) were collected in both waves two and three of the MCS, although the focus of this paper is on data from the latter study (MCS3) at which time index child and their siblings will have had five years of life together.

We explored group differences between the nearest-in-age older siblings (aged five to 15 years) of those MCS cohort member children identified as not having (*n* of siblings *=* 7246) or having (*n* of siblings *=* 257) ID. Those siblings of children who could not be classified as having or not having ID were not included in the analysis. For this study, cohort children with ID were identified using a grouping variable adopted in previous research (Totsika et al. 2018). To identify cohort children with ID, data from the second, third and fourth waves of the MCS were used through a four-step process. At age seven, trained interviewers assessed children’s word reading and pattern construction skills, two scales from the British Ability Scales (BAS-II; Elliott et al. 1996) along with mathematics ability (NFER Progress in Maths). A factor analysis of the age standardized scores of these measures provided a total cognitive ability index *g* that accounted for 63% of the total variance across these measures. Intellectual disability was defined as a score two standard deviations below the mean of the total cognitive ability index. A total of 352 children were classified as having ID (g scores <70) using these age seven variables. Age seven was selected as the age of first choice to identity ID because cohort children would be around two years into their formal education in the UK and at an age when identifying children with ID is arguably ideal (Maulik et al. 2011). For those children unclassified at age seven (e.g., because test data were missing or their family did not respond to MCS at wave four), cognitive test data (BAS-II; Elliott et al. 1996) at age five were used in a similar way. This second step identified a further 137 children with ID. For children that remained unclassified following the first two steps, age-standardized scores on the Bracken School Readiness Assessment –Revised (Bracken 1998) at age three were used. A fourth step used parent and teacher reported information at age seven. Where both parent and teacher had independently indicated that the cohort child had special education needs, and additionally the teacher reported that the cohort child was performing significantly below average on five academic outcomes associated with reading, writing and maths, then the child was classified as having an ID. A further 17 children were identified as having ID at this step.

The four step classification process resulted in 555 cohort children being identified as having ID from a total sample of 19,244 MCS children (equivalent to 2.7% weighted, or 2.9% unweighted, of the MCS sample). Of these MCS children identified as having ID, 257 had one older sibling aged 5–15 at wave three with suitable data available for analysis. Although information was provided on more than one older sibling where applicable, we only included one available older sibling, nearest in age to the cohort member, in the analysis.

### Participants

Demographic characteristics of the identified older siblings of children with ID and children without ID are summarized in Table 1. There are no statistically significant group differences in these characteristics for older siblings of children with and without ID for the following measures: the older sibling being male; older sibling and cohort member being the same sex; the age of the cohort member; and the age difference between the older sibling and the main cohort member. Siblings of children with ID were more likely to be living in a single parent household (χ^2^(2, *N* = 7783) = 27.80, *p* < 0.001), be from a family who experienced more socio-economic deprivation (χ^2^(1, *N =* 7763) =102.08, *p* < 0.001), and have a primary parent or carer experiencing psychological distress (χ^2^(1, *N* = 7458) = 6.27, *p* = 0.013). In addition, older siblings of children with ID were more likely to have a younger brother or sister (the main cohort member) with elevated SDQ (Strengths and Difficulties Questionnaire; Goodman 1997) total difficulties scores (χ^2^(1, *N* = 5186) = 127.57, *p* < 0.001). The number of children in the household was higher for households of a child with ID (*t(1, 389)* = −5.88, p < 0.001). In addition, siblings of children with ID were more likely to be older (*t(1, 389)* = −2.90, *p* = 0.004).

**Table 1:** Group differences for demographic and family factors for older siblings of children with or without ID (results weighted)

Demographic and family factors	Siblings of children without ID [95% CI]	Siblings of children with ID [95% CI]	χ^2^	*p*
Older sibling male	51.2% [49.6, 52.7]	51.9% [45.3, 58.4]	0.05	0.826
Household experiencing socio-economic deprivation	44.0% [41.8, 46.3]	77.6% [70.8, 83.3]	102.08	<0.001
Siblings are same sex	50.0% [48.4, 51.6]	46.0% [36.5, 55.8]	0.78	0.426
Single parent household	15.9% [14.8, 17.1]	28.9 [23.2, 35.4]	27.80	<0.001
Main carer experiencing psychological distress	3.3% [2.9, 3.9]	6.7 [4.0, 10.9]	6.27	0.013
Cohort child with or without ID having an SDQ total difficulties score in the “abnormal” range	4.3% [3.6, 5.0]	27.8% [19.5, 38.0]	127.57	<0.001
Primary respondent was natural parent of cohort member	99.6% [99.3, 99.8]	100.0% [100.0, 100.0]	0.49	0.953
Primary respondent was female	97.9% [97.4, 98.4]	97.8% [93.5, 99.3]	0.01	0.928
	Siblings of children without ID mean (SD)	Siblings of children with ID mean (SD)		*p*
Number of children in the household	2.83 (1.48)	3.31 (1.26)		<0.001
Age of older siblings	9.41 (3.47)	10.02 (3.29)		0.004
Age of cohort member children	4.80 (0.58)	4.82(0.46)		0.770
Age difference between older sibling and cohort member	3.68 (2.74)	3.85 (2.89)		0.515
Age of primary respondents	35.21 (9.57)	33.39 (6.19)		0.002

*df* for each *test* = 1, 389 with the exception of: Age of cohort member children; Age difference between older sibling and cohort member; and Age of primary respondents *df* = 1, 387; Single parent household *d*f = 2, 773; and Primary respondent was natural parent of cohort member *df* = 3, 1221

The primary respondent was usually the natural parent of the cohort member child (for 99.6% of cohort members) and was typically the mother (97.9% of primary respondents were female) for both those families with and without a child with ID. Primary respondents of a cohort member with ID were younger (mean age = 33.39; SD =6.19; Range = 26 compared to the primary respondents of a cohort member without ID (mean age = 35.21; SD =9.57; Range = 42 (*p* = 0.002). It was not possible to determine if the older siblings themselves had an intellectual disability from the data available from the MCS for this secondary data analysis. There was also no genetic information available for siblings, including no report of autism or other diagnoses for siblings.

### Measures

#### Behavioral and Emotional Adjustment

The behavioral and emotional adjustment of siblings was measured using the Strengths and Difficulties Questionnaire (SDQ; Goodman 1997). Main respondents were asked to complete the SDQ about the older sibling. The SDQ includes 25 items to assess the psychological adjustment of young people and children (Goodman 1999) using a three-point rating scale (i.e. ‘not true’, ‘somewhat true’ or ‘certainly true’) to assess the extent to which the statement applies to the child. Items include statements such as the older sibling being “considerate of other people’s feelings” and “often unhappy, down-hearted or tearful”. The items represent five distinct scales: emotional symptoms, conduct problems, hyperactivity/inattention, peer relationship problems, and prosocial behavior. A SDQ total difficulties score is derived by summing the first four sub-scores (excluding prosocial behavior). For the purposes of this study, SDQ scores were also dichotomized at the advised clinical cut off levels (Goodman 1999) for normal and “abnormal” scores. Primary respondents also completed the SDQ for the cohort child (the MCS cohort child with or without ID).

#### Primary Carer Psychological Distress

To measure the psychological distress of the primary respondent, usually the biological mother, MCS used the K6 self-completion measure (Kessler et al. 2002). The K6 asks the person completing it how often they have felt in the last 30 days “nervous”, “hopeless”, “restless”, “fidgety”, “so depressed that nothing could cheer you up” and whether everything was an “effort and worthless”. Responses are based on a five point rating scale to measure the extent to which each question applies to the respondent. These items are summed to derive a 0 to 24 score. For the purposes of the present study, this scale was dichotomized with primary carers scoring 13 and above (which has been identified as a reliable cut-off for identifying serious mental illness) versus below a score of 13 (Furukawa et al. 2003; Kessler et al. 2010).

#### Socio-Economic Deprivation

To measure the socio-economic deprivation experienced by MCS families at wave three, a composite variable was produced. This variable was based on previous research (Totsika et al. 2011, 2013, 2014, 2015). Five indicators were incorporated to form this composite measure. Subjective poverty was measured on a five point rating scale identifying how well families felt they were managing financially. Responses from primary respondents were dichotomized into families finding it “quite” or “very difficult” to manage financially versus families who were managing financially. MCS3 included five items about material deprivation by enquiring if families had access to basic material goods relevant in the UK, such as a weatherproof coat for the cohort member, two pairs of all-weather shoes, or if they were able to afford an annual holiday without staying with relatives. Older siblings’ families were grouped into those who could not afford two or more of those items versus those families that could afford all or all but one of those items. The economic activity of families was dichotomized by workless families versus families with at least one parent or carer working. Income poverty was measured using the OECD’s definition: families with an income below 60% of UK median equivalized income versus families with an income above this level. Neighborhood deprivation was measured using the UK Indices of Multiple Deprivation (IMD) information which measure deprivation for small geographical areas based on seven different domains of deprivation derived from national Census data. This includes measures of income, employment, education, health, crime, housing, and environment (Gill 2015). Neighborhood deprivation rankings for England, Northern Ireland, Scotland and Wales were incorporated into one variable and dichotomized by whether families lived in a neighborhood in the bottom (most deprived neighborhoods) quintile versus all other quintiles for their UK country.

The five dichotomized indicators were summed into one socio-economic deprivation composite measure. The composite measure values ranged from zero to five with higher values relating to higher levels of deprivation for families. This summed scale was then further dichotomized to identify those families experiencing socio-economic deprivation (those with one or more indicators of socio-economic deprivation) versus those families experiencing no indicators of socio-economic deprivation. This dichotomization was used because almost 50% of families had no indicators of socio-economic deprivation, and so the summed score had a highly skewed distribution that was otherwise difficult to transform.

### Procedure and Analysis Approach

The data for this study were from MCS3, available from the Centre for Longitudinal Studies, UCL: IOE (“Millennium Cohort Study: Third Survey,” 2017). Data were available to download from the UK Data Service. Ethical approval for MCS1 was gained by the original investigators from the South-West Multi-Centre Research Ethics Committee and MCS2 and MCS3 was gained from the London Multi-Centre Research Ethics Committee. All adult respondents provided informed, written consent to take part in the MCS study for their own involvement and also as parent/guardian of the participating child/children. To download the data, researchers must register and agree to a number of data privacy conditions, including maintaining the confidentiality and anonymity of the families included in the study. In addition, for the present secondary data analysis, ethical approval was granted from the University of Warwick’s Humanities and Social Sciences Research Ethics Committee as per institutional requirements. Analysis was performed using SPSS version 24©. The sample weightings required to ensure the sample was representative of the UK population meant that all analysis was performed through SPSS complex samples options (Jones and Ketende 2010; IBM Software Group 2012).

Our first research objective was to explore whether there were group differences in the SDQ total difficulties score and SDQ sub-scale scores between those older siblings of cohort member children with ID and those older siblings of cohort children without ID. This was explored and analyzed using t-tests through general linear models. Group differences in the proportion of siblings scoring in the abnormal range on SDQ scores were also explored using Odds Ratios. By exploring this question in both ways we were able to not only explore general group differences, but also to consider the differences between those siblings with more concerning levels of behavioral and emotional problems (i.e. those scoring above the clinical cut off for scores on the SDQ).

We then explored whether any group differences would remain once socio-economic, demographic, and family factors were controlled and which of these factors were associated with siblings’ behavioral and emotional adjustment. Using dichotomous scores for SDQ domains (abnormal range vs. not) as outcomes, logistic regression models were fitted to examine group differences alongside the following factors: sex of the older sibling, number of siblings and cohort member(s) in the household, age difference between cohort member and older sibling, the age of the older sibling, same or different sex for sibling pairs, family socio-economic deprivation, single parent household, primary carer psychological distress (typically maternal psychological distress; K6), and the behavioral and emotional adjustment of the MCS cohort member child (SDQ total behavior problem score). Logistic regression was selected rather than ANCOVA to explore the second research question as we were interested in correlates of older siblings’ behavioral and emotional adjustment and specifically in those older siblings experiencing elevated SDQ scores who may represent the most important at-risk group of siblings from a clinical perspective.

## Results

### Sibling Behavioral and Emotional Adjustment Group Differences

Mean scores for primacy carer SDQ ratings of siblings of children with ID and without ID are presented in Table 2. The SDQ total problems (*t(1, 389)* = −2.97, *p* = 0.003, *d* = 0.19*)*, peer problems (*t(1, 389)* = −3.85, *p* < 0.001, *d* = 0.26) and conduct problems (*t(1, 389)* = −3.46, *p* = 0.001, *d* = 0.22) scores were higher for older siblings of children with ID. Cohen’s *d* estimates indicate small to very small effect size differences between the groups. No statistically significant group differences were found for hyperactivity (*t(1, 389)* = −1.65, *p* = 0.101, *d* = 0.09), prosocial behavior (*t(1, 389)* = 1.48, *p* = 0.139, *d* = 0.09), and emotional problems (*t(1, 389)* = −0.68, *p* = 0.495, *d* = 0.05).

**Table 2:** SDQ mean scores for siblings of children with ID and without ID (results weighted)

SDQ score	Non ID mean (SE)	ID mean (SE)	ID mean difference [95% CI]	*t*	*p*	Cohen’s *d*
Total behavior problems	7.48 (0.10)	8.98 (0.49)	−1.50 [−2.50, −0.51]	−2.97	0.003	0.19
Peer problems	1.42 (0.03)	2.01 (0.15)	−0.59 [−0.89, −0.29]	−3.85	<0.001	0.26
Conduct problems	1.54 (0.03)	2.03 (0.14)	−0.49 [−0.77, −0.21]	−3.46	0.001	0.22
Emotional symptoms	1.79 (0.03)	1.93 (0.19)	−0.14 [−0.53, 0.25]	−0.68	0.495	0.05
Hyperactivity/inattention	2.77 (0.04)	3.05 (0.17)	−0.29 [−0.63, 0.06]	−1.65	0.101	0.09
Prosocial	8.49 (0.02)	8.31 (0.12)	+0.18 [−0.06, 0.43]	1.48	0.139	0.09

*df* for each *test* = 1, 389

Table 3 presents Odds Ratios for comparisons of older siblings of children with and without ID in terms of SDQ scores above the abnormal range cut-offs. The siblings of children with ID were more likely to have elevated SDQ scores than the siblings of children without ID for total problems (*OR* = 1.65; 95% CI 1.04, 2.62; *p* = 0.031), peer problems (*OR* = 2.01; 95% CI 1.37, 2.95; *p* < 0.001), and conduct problems (*OR* = 1.75*;* 95% CI 1.19, 2.57; *p* = 0.004). No statistically significant group differences were found for elevated levels of hyperactivity (*OR* = 0.65*;* 95% CI 0.33, 1.30; *p* = 0.217), limitations in prosocial behavior (*OR* = 1.23; 95% CI 0.85, 1.79; *p* = 0.269), and emotional problems (*OR* = 1.21; 95% CI 0.70–2.10; *p* = 0.500).

**Table 3:** Group differences for elevated SDQ scores (results weighted)

	Siblings of children without ID abnormal score [95% CI]	Siblings of children with ID abnormal score [95% CI]	*OR*	*OR* 95% CI
SDQ total behavior problems	8.9% [8.1, 9.7]	13.9% [9.4, 20.1]	1.65	1.04, 2.62
Peer problems	11.6% [10.8, 12.6]	20.9% [15.3, 27.8]	2.01	1.37, 2.95
Conduct problems	12.0% [11.0, 13.1]	19.2% [14.0, 25.8]	1.75	1.19, 2.57
Emotional problems	10.3% [9.6, 11.2]	12.2% [7.6, 19.2]	1.21	0.70, 2.10
Hyperactivity	7.1% [6.4, 7.8]	4.7% [2.4, 8.9]	0.65	0.33, 1.30
Prosocial	15.8% [14.9, 16.8]	18.8% [13.8, 25.2]	1.23	0.85, 1.79

*df* for each *test* = 1, 389

### Logistic Regression Analyses

The results of the logistic regression models are summarized in Table 4. Logistic regression models were used to predict elevated (“abnormal” range) scores for each of the five SDQ sub scores as well as the total difficulties score. For each of these six logistic regression models, socio-economic deprivation, elevated behavior problems for the MCS cohort member child (i.e., whether the cohort child’s SDQ total score was in the abnormal clinical range), and the older sibling being male were statistically significant predictors of whether older siblings had elevated SDQ scores. Primary carer psychological distress, and being from a single parent household were also statistically significant predictors in most of the regression models.

**Table 4:** Logistic regression models showing correlates (odds ratios and 95% CIs) of elevated SDQ total behavior problems score and sub-scores (results weighted)

	Elevated total problems score *OR* [95% CI]	Peer problems *OR* [95% CI]	Conduct problems *OR* [95% CI]	Prosocial behavior *OR* [95% CI]	Hyperactivity/ inattention *OR* [95% CI]	Emotional symptoms *OR* [95% CI]
Cohort child has intellectual disability	0.88 [0.41, 1.89]	0.94 [0.49, 1.77]	0.83 [0.42, 1.66]	0.88 [0.44, 1.76]	0.30 [0.10, 0.87]*	0.67 [0.34, 1.35]
Older sibling being male	2.03 [1.55, 2.65]***	1.74 [1.39, 2.17]***	1.59 [1.26, 2.00]***	2.23 [1.82, 2.73]***	2.93 [2.15, 4.00]***	0.80 [0.64, 1.00]*
Socio-economic deprivation	1.90 [1.44, 2.51]***	1.63 [1.27, 2.09]***	1.75 [1.39, 2.21]***	1.33 [1.11, 1.60]**	1.53 [1.13, 2.07]**	1.44 [1.12, 1.84]**
Cohort child - elevated SDQ total problems score	3.29 [2.17, 4.99]***	3.28 [2.30, 4.70]***	2.48 [1.65, 3.72]***	1.55 [1.00, 2.40]*	2.27 [1.35, 3.82]**	2.73 [1.86, 4.03]***
Single parent household	1.70 [1.23, 2.36]**	1.53 [1.14, 2.04]**	1.71 [1.31, 2.23]***	1.29 [0.99, 1.67]	1.76 [1.23, 2.51]**	1.38 [1.03, 1.87]*
Primary carer experiencing psychological distress	2.47 [1.46, 4.18]**	2.19 [1.27, 3.75]**	2.48 [1.56, 3.95]***	1.28 [0.80, 2.04]	1.64 [0.81, 3.28]	2.61 [1.61, 4.24]***
Older sibling age	0.69 [0.49, 0.98]*	0.89 [0.66, 1.20]	0.79 [0.60, 1.03]	0.95 [0.73, 1.24]	0.83 [0.58, 1.18]	0.77 [0.56, 1.06]
Number of siblings and cohort children in household	0.99 [0.82, 1.20]	0.98 [0.83, 1.16]	0.89 [0.76, 1.05]	0.84 [0.73, 0.97]*	1.10 [0.91, 1.34]	1.11 [0.90, 1.35]
Older sibling and cohort child being the same sex	0.85 [0.65, 1.11]	1.08 [0.85, 1.36]	0.97 [0.78, 1.21]	1.04 [0.84, 1.29]	0.92 [0.69, 1.21]	0.97 [0.78, 1.20]
Age difference between cohort child and older sibling	1.40 [0.99, 1.96]	1.07 [0.80, 1.45]	1.17 [0.90, 1.52]	0.93 [0.72, 1.21]	1.18 [0.84, 1.66]	1.35 [0.98, 1.86]

*df* for each predictor in the logistic regression models = 1, 386

*p ≤ 0.05; ** *p* < 0.01; ***p < 0.001

After taking into account all other correlates, sibling group membership (siblings of children with/without ID) was not associated with older siblings’ SDQ scores in five of the six regression models. The exception was for SDQ hyperactivity scores. The odds ratio for older siblings of children with ID having raised hyperactivity levels was 0.30 *(p* = 0.027; 95% CI 0.10, 0.87), indicating that the odds of them having increased hyperactivity levels was lower than for siblings of children without ID after accounting for other factors.

## Discussion

The present study explored whether there were group differences in behavioral and emotional adjustment between older siblings of children with and without ID using a UK population-representative sample. In univariate analyses, statistically significant group mean differences were found for some, although not all, domains of the SDQ (total problems, peer problems, and conduct problems), where siblings of a child with ID had more problems than their peers. Effect sizes for these group differences were small. Using Odds Ratios to examine the proportion of each group meeting clinical cut-off scores on SDQ domains, older siblings of children with ID were approximately 1.5—2 times more likely to have problems in the abnormal range on three SDQ domains (total problems, peer problems, and conduct problems) but did not differ on the other three SDQ domains. These results show that older siblings of children with ID have increased (small effect sizes) total problems, peer and conduct problems compared to older siblings who do not have a brother or sister with ID. Although some older siblings of children with ID have elevated SDQ scores compared to siblings of children without ID, differences in clinical levels of behavioral and emotional adjustment problems relate to a small group of older siblings with particularly poor adjustment.

Once additional factors, informed by existing literature, were included in logistic regression models, the ID group variable was not significantly associated with sibling behavioral and emotional adjustment except for one SDQ domain (hyperactivity). These findings suggest that older siblings of children with ID are not at an increased risk of behavioral and emotional adjustment problems due simply and directly to having a brother or sister with ID. These findings are largely consistent with Emerson and Giallo’s (2014) and Neely-Barnes and Graff’s (2011) research exploring sibling outcomes in non-UK national samples and focused on mixed disability groups.

The demographic and family factors included in Table 1 indicate that older siblings with a brother or sister with ID were more likely to be from a family experiencing socio-economic deprivation, a single parent household, for their primary parent/carer to be experiencing psychological distress, and their MCS cohort brother/sister had elevated behavioral and emotional problems. These variables are risk factors for poorer well-being for children in general. In addition, older siblings with a brother/sister with ID were older themselves, lived in larger families, and had younger primary carers compared to other older siblings.

In the logistic regression models, being a male older sibling, coming from a family experiencing socio-economic deprivation, living in a single parent household, having a brother/sister with elevated behavioral and emotional problems, and having a primary carer with high levels of psychological distress were all consistently and independently associated with sibling emotional and behavioral adjustment. Therefore, an array of family and social factors in particular were associated with sibling adjustment (cf. Kovshoff et al. 2017). Accounting for these variables, and other demographic factors, reduced the initial sibling group differences to be outside of the range of statistical significance. The findings extend those from previous research beyond a focus on socio-economic factors to broader family systems issues and reinforce the importance of considering sibling adjustment from a multi-layered systems perspective.

In the logistic regression models, older siblings of children with ID were found to be less likely to be identified as hyperactive/inattentive compared to those siblings whose brother or sister did not have ID. In previous MCS research, data show that the children with ID in the sample were also more likely to be hyperactive (Totsika et al. 2011), and in this sample where the cohort children have an older sibling, 27.5% of children with ID are identified as having elevated hyperactive behaviors compared to 5.4% of those cohort children without ID. This contrast may help to explain the current findings. It is possible that primary caregivers may have completed the measure of the older siblings’ hyperactivity in the context of the behavior of their younger brother or sister with ID. Parents therefore may have been indicating that, relative to their brother or sister with ID, the older sibling was less hyperactive. To explore this further, and other potential explanations of this finding, in future research, it would be particularly useful to gather data from multiple informants (e.g., the older sibling themselves and possibly their class teacher). It is also possible that this finding may have been a Type I error due to multiple testing of the dataset, and this should be examined further in additional research.

How do findings from the present study help address the questions of whether, and how, siblings of children with ID might be at increased risk for behavioral and emotional problems? First, it is clear that these siblings are an at-risk group. The current population-based sample confirms this, and although the increased risk is 1.5—2 times that for other older siblings, only a minority of siblings of children with ID may experience (up to approximately 20% - see Table 3) elevated problems of behavioral or emotional adjustment. Second, the regression models suggest that a range of social and family factors are associated with sibling adjustment. Most of these putative risk factors (Table 1) also occur at higher levels in families of children with ID. However, given the cross-sectional design, we cannot distinguish how these factors may affect sibling adjustment. One hypothesis is that there is no effect of child ID on their siblings’ behavioral and emotional adjustment but other social and family variables determine sibling adjustment. An alternative hypothesis is that growing up with a brother or sister with ID indirectly affects siblings’ adjustment by directly increasing other risks (e.g., exposure to poverty, carers with psychological problems, and a brother or sister who also has behavioral and emotional problems). These alternatives should be explored in future research, especially longitudinal research that can establish causal pathways for siblings’ behavioral and emotional adjustment.

Existing literature has explored a number of other demographic variables (the siblings being of the same or different sex, sibling age, and the age difference between the older sibling and the child with ID) as correlates of siblings’ behavioral and emotional adjustment (Cuskelly and Gunn 2006; Ivey and Barnard-Brak 2009; Martin and Horriat 2012; Burke et al. 2012; Goudie et al. 2013; Walton 2016). However, these factors did not emerge as significant predictors in the logistic regression models in the present study. This may have been because the data focused only on older siblings, or because these factors are more important within families of children with disabilities rather than for siblings generally. Existing population based studies exploring sibling differences included not just older siblings, but both older and younger siblings (Goudie et al. 2013; Emerson and Giallo 2014; Neely-Barnes and Graff 2011). Furthermore, the cohort children in this sample were all young. It is foreseeable that as children with ID age their behavior may be perceived as more challenging and this may have a greater effect on family members, including siblings.

Although a strength of the current study is the population-based nature of the sample, a key limitation of the present study is that it only includes older siblings. There are some data to suggest that birth order may have an impact on various elements of sibling experience. Saxena (2015) highlighted that in adulthood, older siblings have been found to be more involved in care and suggest this may be in response to parental expectations of older siblings in childhood. There are also data to suggest that relationships between siblings and their brother or sister with developmental disabilities may be more positive when they are the older sibling (Orsmond et al. 2009). Therefore, the current findings may not apply to the behavioral and emotional adjustment of younger siblings.

There are a number of limitations related to the nature of doing a secondary analysis, in that any analysis is limited by the variables made available. For example, it was not possible to identify if the older siblings themselves had ID or any other disability or genetic difference. Related to the population-based nature of the sample, the ID grouping variable is likely to include mostly children with mild to moderate ID. Using a population based sample from a national birth cohort study meant that children with rare disorders or severe to profound ID would have been missed from sampling or assessment processes (because of very low population prevalence). Further research is needed to explore sibling behavioral and emotional adjustment when they have a brother or sister with severe ID and/or identified genetic syndromes. In addition, only one measure (the SDQ) was used to explore sibling outcomes. The SDQ is a screening measure for mental health rather than a more complete measure of sibling psychological adjustment. Future research should include a broader range of outcome measures for siblings. A final limitation is that the data in this analysis were based on primary carer reports of sibling adjustment and also of all other study variables. Thus, there is a problem of source variance. In future research, multi-informant methods, including sibling self-reports, are also needed (Kovshoff et al. 2017).

In terms of practical implications, policy makers and practitioners may want to concentrate support on siblings of children with ID who are considered at greater risk of other adversities – such as socio-economic hardships, high levels of primary carer psychological distress, and where their brother or sister with ID has significant behavioral and emotional problems. There is more work to be done on a structural level to address socio-economic inequalities or through specifically targeted family interventions to support these more-at-risk siblings and their families.
